# Behavioral differences at scent stations between two exploited species of desert canids

**DOI:** 10.1371/journal.pone.0232492

**Published:** 2020-05-15

**Authors:** Maksim Sergeyev, Kelsey A. Richards, Kristen S. Ellis, Lucas K. Hall, Jason A. Wood, Randy T. Larsen

**Affiliations:** Department of Plant and Wildlife Sciences, Brigham Young University, Provo, UT, United States of America; Institute of Zoology, CHINA

## Abstract

Coyotes (*Canis latrans*) and kit foxes (*Vulpes macrotis*) are desert canids that share ecological similarities, but have disparate histories with anthropogenic pressure that may influence their responses towards novel stimuli. We used remote cameras to investigate response to novel stimuli for these two species. We predicted that coyotes (heavily pressured species) would be more wary towards novel stimuli on unprotected land (canid harvest activities are permitted) than in protected areas (canid harvest activities are not permitted), whereas kit foxes (less pressured species) would exhibit no difference. We examined differences in the investigative behaviors at 660 scent stations in both protected and unprotected areas. Coyotes showed no differences between protected and unprotected land and were generally more wary than kit foxes, supporting our prediction. Kit foxes were more investigative on protected land, contrary to our expectations. Our study provides evidence that anthropogenic pressure can alter the behaviors of wildlife species.

## Introduction

Behavioral responses of wildlife to novel anthropogenic objects vary greatly and can be influenced by social status, trophic level, past experiences with anthropogenic stimuli, and differences in personality [1–4]. Responses to novel objects are generally categorized as either neophilic or neophobic. Neophilia (attraction to novelty) can be an advantageous behavior in discovering new resources, related to the concept of boldness (tendency to take risks), however, increased conflict with humans may arise as animals interact with anthropogenic stimuli [4, 5]. Conversely, neophobia (fear of novel stimuli) is typically classified as gustatory (novel food sources), social (novel interactions between conspecifics), or predator [novel objects perceived as predatory threats; 2] Neophobia has been associated with lower trophic levels and social status, and can be influenced by familiarity with surroundings [1, 2, 6]. Repeated exposure to anthropogenic stimuli may cause habituation (decreased sensitivity to novel objects) or sensitization [increased avoidance; 4]. Consequently, prior interactions with anthropogenic disturbances can influence behavioral responses to novel stimuli.

Species subjected to intense anthropogenic pressure (e.g., hunting, trapping) may exhibit increased wariness than less pressured species [7–9]. If behaviors that render individuals susceptible to hunting and trapping by humans (e.g., investigating anthropogenic stimuli) have a genetic basis, these behaviors would be subjected to selection [10]. Thus, pressure towards hunted and trapped species could reduce the genetic availability of specific behaviors (that increase mortality) and, over generations, influence interactions with novel anthropogenic stimuli [9]. As a result, species with a history of anthropogenic pressure may exhibit increased neophobia.

Coyotes (*Canis latrans*) and kit foxes (*Vulpes macrotis*) are two canid species found across arid environments of North America [11, 12] that have ecological similarities but disparate histories of anthropogenic pressure that may influence their behaviors [13]. Coyotes, long considered a nuisance species, have been subjected to intense lethal control [14–19], potentially causing heightened neophobia [6, 20–29]. Alternatively, kit fox populations have declined in past decades and have been the focus of conservation efforts by state and federal agencies [30–34]. While kit foxes were historically trapped and hunted, they were not subjected to intense exploitation and targeted removal as were coyotes. Kit foxes are generally less wary than coyotes [23, 35] and are innately investigative towards novel stimuli [36–40], consistent with the a species that has experienced less intense exploitation.

Anthropogenic pressure may influence behavior of coyotes and kit foxes differently in areas where hunting and trapping occur compared to areas where they are prohibited [9]. We evaluated behavioral differences between coyotes and kit foxes to novel stimuli at 660 scent stations across Utah in areas with and without anthropogenic pressure. We predicted that 1) kit foxes would be more investigative than coyotes in general and 2) coyotes would be less investigative towards novel stimuli in unprotected areas than protected areas, whereas kit foxes would exhibit no difference.

## Methods

### Ethics statement

Fieldwork was approved and sanctioned by United States Department of Defense (DoD) and Utah Division of Wildlife Resources and conducted in compliance with the Institutional Animal Care and Use Committee of Brigham Young University.

### Study areas

**Fig 1:**
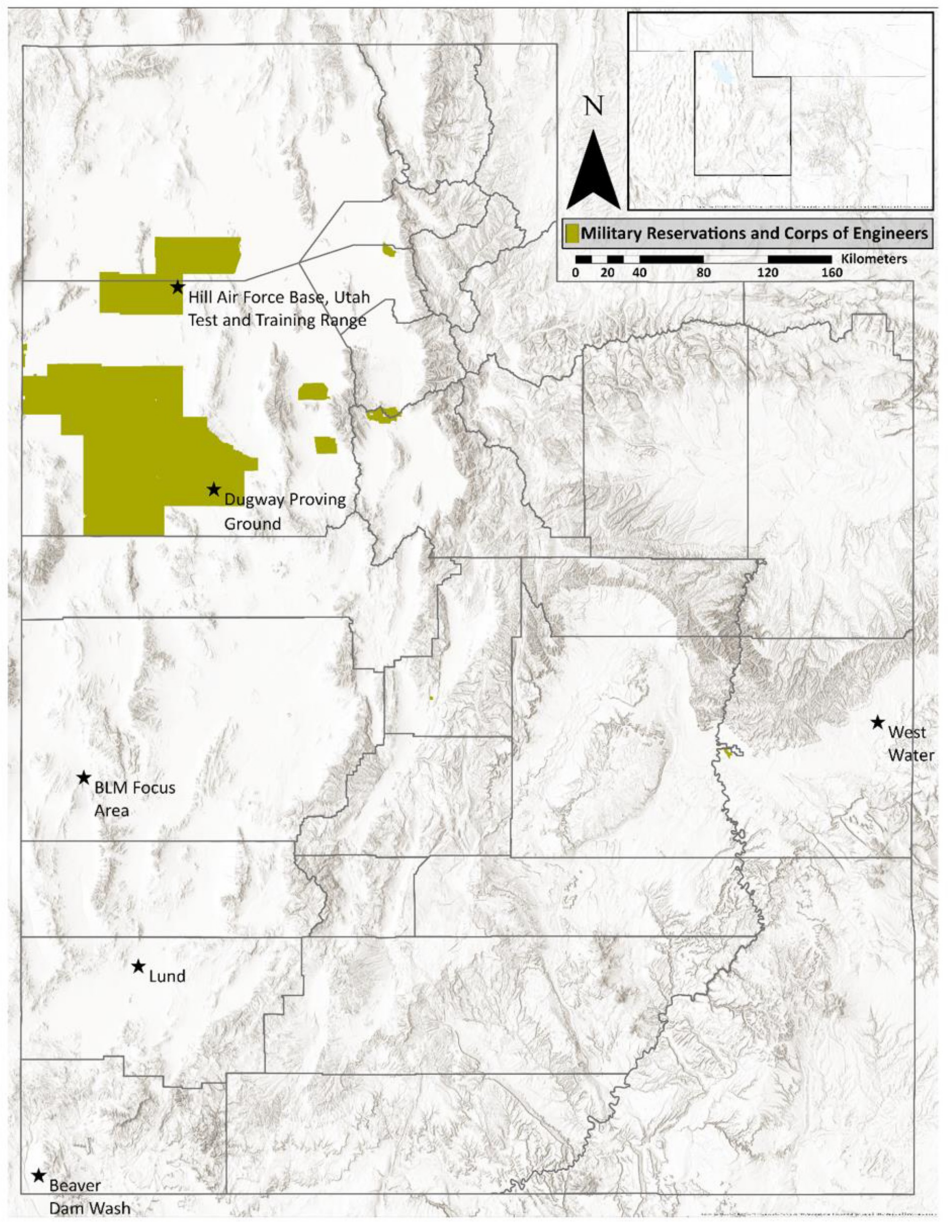
Map of study area comparing behavioral differences at scent stations between coyotes and kit foxes throughout Utah, USA. Black stars indicate sampling areas for camera grid. Protected areas are denoted with green shading and used for comparison of behavior between areas with hunting/trapping and without.

We conducted our research at nine study areas across southwestern Utah, U.S.A. (Fig 1), where coyotes and kit foxes are sympatric [41]. Sites were located in the West desert and throughout the southern half of the state. Study areas were in arid landscapes, however, climatic conditions varied between sites. Two study areas were on DoD land where hunting and trapping was prohibited. We considered DoD areas “protected”, whereas remaining areas were on public land and allowed hunting and trapping.

### Data collection

To monitor the behavior of canids, we created a grid of sample cells with forced minimum distance of 4 km between cells [42] except on military test ranges (due to safety concerns and site-specific protocols, forced minimum distance was restricted to 1.61 km). A 2.6 km radius buffer was used around each point. We selected this distance based on the square root of home ranges for coyotes occupying similarly semi-arid environments as it reflects daily movement [43–45]. We deployed scent stations between May 2015 and October 2016. To promote independence, we placed cameras within a 300 m buffer of the cell’s centroid. Scent stations consisted of an infrared camera (Reconyx^©^ PC900) attached to a post, positioned 27 cm above ground. Cameras were motion-activated and captured images when movement was detected. We randomly assigned every station one of three possible novel objects: pre-scented plaster of Paris tablet with fatty acid lure (Pocatello Supply Depot, Pocatello, Idaho), bundle of nine cotton swabs, or a hollowed golf ball mounted on a wooden dowel. Cotton swabs and golf balls were impregnated with Red and Gray Fox or Willey liquid lure (Murray’s Lures, Walker, West Virginia, USA). Attractants were positioned two meters from the camera. Prior research showed no difference in detection between objects or species [46]; additionally, objects were randomly assigned to avoid bias. Scents were refreshed after one week and monitored for an additional week. We recognize the potential influence that vegetation has on the behavior of wildlife, thus, we accounted for differences in vegetation using Landscape Fire and Resource Management Planning Tools (LANDFIRE) data provided by U.S. Forest Service and U.S. Department of the Interior [47]. We classified vegetation as barren (16%), shrub (67%), exotic herbaceous (13%), conifer (2%), or unknown (2%).

**Fig 2:**
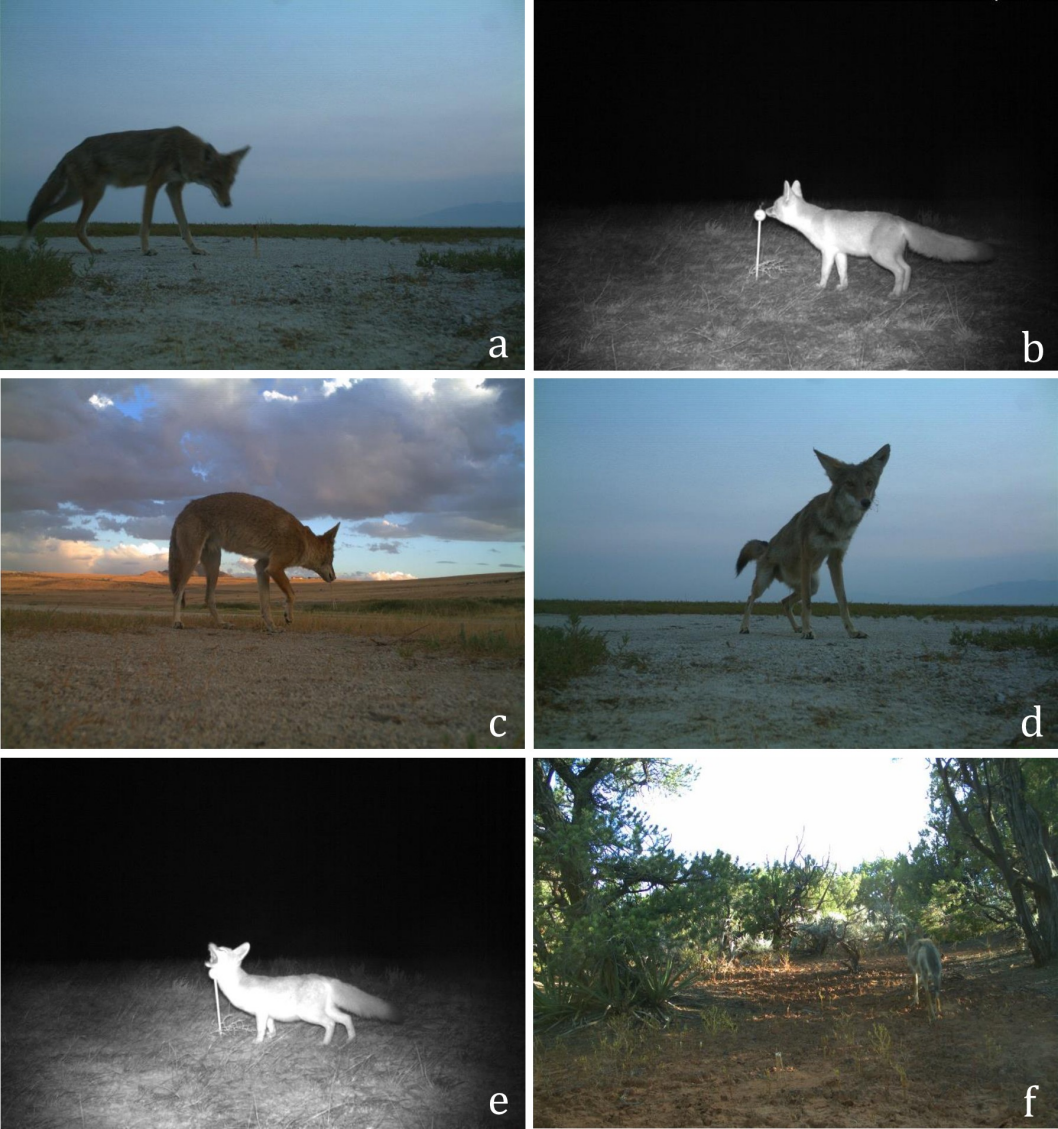
Ethogram of behaviors investigated in this study. From top left to bottom right, panel A shows a coyote approaching the lure; in panel B, a kit fox is sniffing the lure; panel C shows a coyote biting the lure; in panel D, a coyote is urinating on the lure; in panel E, a kit fox is rubbing against the object. Panels A-E are examples of the different behaviors categorized as investigative. In panel F, a coyote is near the lure but not interacting with it (classified as non-investigative).

To analyze canid behavior, we initially separated photographs by species and classified proximity to stimulus as close (within a one meter) or far. We then classified behavior as investigative or non-investigative. Photographs were considered investigative when behaviors conveyed attention toward the stimulus (scented object or camera; Fig 2). Investigative behaviors included approaching, sniffing or biting the object, or scent marking by urinating or rubbing against the object. Photographs were considered non-investigative when animals displayed no attention to stimuli but remained within the field of view (Fig 2). Repeated visits may have led to increasingly investigative behavior. However, we were unable to identify individuals and subsequent photos showing investigative behavior would have also been included in the analysis. To ensure consistency when categorizing behavior, one technician first categorized photographs as close or far and another technician categorized photographs as investigative or non-investigative. All processing of photographs was conducted by individuals familiar with the study design and trained to identify photographs by proximity or behavior; photographs were randomly selected to validate classifications.

### Statistical analysis

We used mixed-model linear regression to determine the behavior of canids toward novel stimuli. We used proportion of investigative photographs and proportion of close photographs as separate response variables and evaluated the same set of twelve *a priori* models for both responses (Table 1). We accounted for variation across study areas using random effects in the lme4 package [48] in Program R [49]. We evaluated candidate models using conditional Akaike’s information criterion (cAIC), which is appropriate for evaluating relative fit among mixed-effects models [50, 51]. To evaluate significance of covariates, we examined overlap in 85% confidence intervals around mean estimates [52].

**Table 1:** Model selection comparing proportion of close photographs (A) and investigative photographs (B) of kit foxes and coyotes at scent stations throughout Utah, USA. Top models suggested differences between species and between protected versus unprotected land. Table contains conditional Akaike Information Criteria (cAIC) and Akaike model weight (w_*i*_) and conditional log-likelihood (LL) of candidate models.

	Model	LL	K	cAIC	ΔcAIC	*w*_*i*_
(A)	Species + Protected	-423.10	5	954.35	0.00	0.45
	Species	-424.91	4	955.64	1.29	0.24
	Species * Protected	-422.61	6	955.94	1.59	0.20
	Species + Protected + Vegetation	-421.32	9	959.17	4.82	0.04
	Species + Vegetation	-422.89	8	959.72	5.37	0.03
	Species * Protected + Vegetation	-420.89	10	960.79	6.44	0.02
	Species * Vegetation + Protected	-418.44	13	962.10	7.75	0.01
	Species * Vegetation	-420.10	12	963.25	8.90	0.01
	Protected	-428.46	4	1000.17	45.82	0.00
	Intercept	-429.64	3	1000.28	45.93	0.00
	Vegetation	-427.15	7	1002.48	48.13	0.00
	Vegetation + Protected	-425.90	8	1003.11	48.76	0.00
(B)	Species + Protected	-407.32	5	873.63	0.00	0.52
	Species * Protected	-406.96	6	875.45	1.82	0.21
	Protected	-410.93	4	877.12	3.49	0.09
	Species	-406.32	4	877.25	3.62	0.09
	Species + Protected + Vegetation	-404.72	9	879.09	5.46	0.03
	Intercept	-409.74	3	880.23	6.60	0.02
	Species * Protected + Vegwgetation	-404.42	10	880.93	7.30	0.01
	Species + Vegetation	-405.82	8	881.45	7.82	0.01
	Vegetation + Protected	-408.71	8	883.25	9.62	0.00
	Vegetation	-409.76	7	884.71	11.08	0.00
	Species * Vegetation + Protected	-401.66	13	885.08	11.45	0.00
	Species * Vegetation	-402.76	12	887.69	14.06	0.00

## Results

Coyotes and kit foxes visited 183 of 660 (~28%) scent stations. We recorded 4,142 photographs of both species and identified 1,008 separate visits. Of the total visits, 217 were of coyotes (73% on protected land, 27% unprotected on land) and 791 were of kit foxes (77% on protected land, 23% on unprotected land).

**Fig 3:**
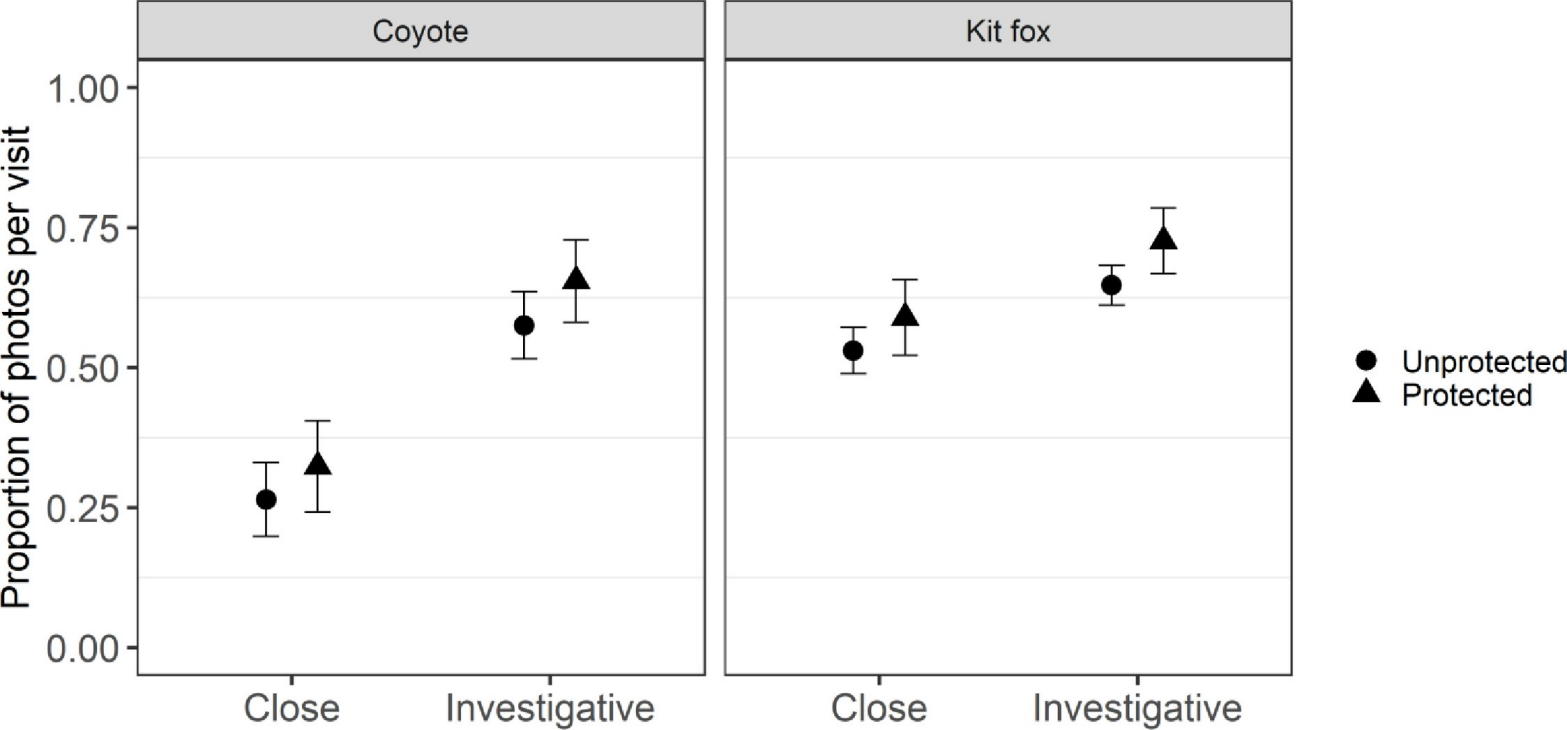
Proportion of investigative and close (within one meter) photographs per visit (± 85% CI) by kit fox (*Vulpes macrotis*) and coyote (*Canis latrans*) at scent stations in Utah (2015–2016). Protected sites were on Department of Defense land, where hunting and trapping were not allowed. Unprotected sites were on public land and permitted harvest activities.

Our results suggested that canid behaviors differed according to species and land ownership. We found strong support for species and protected areas explaining variation in the proportion of close photographs per visit (3 models with ΔcAIC < 4 included combinations of species and protected area fixed effects, combined *w*_*i*_ of these models = 0.90; Table 1A). The most-supported model for proportion of close photographs per visit included additive effects of species and protected areas (*w*_*i*_ = 0.45), and this response (mean ± SE) was greater for kit foxes (0.56 ± 0.02; 85% CI = 0.52–0.60) than for coyote (0.29 ± 0.03; 85% CI = 0.24–0.35; Fig 3), consistent with predictions. Proportion of close photographs per visit was greater on protected areas (0.46 ± 0.03; 85% CI = 0.40–0.51) than on unprotected areas (0.40 ± 0.02; 85% CI = 0.36–0.43) for both species, though 85% confidence intervals overlapped.

Similarly, we found support for differences in proportion of investigative photographs per visits between protected and unprotected areas and species (4 models with ΔcAIC < 4 included combinations of species and protected area fixed effects, combined *w*_*i*_ = 0.91; Table 1B). The most-supported model for proportion of investigative photographs per visit again included additive effects of species and protected areas (*w*_*i*_ = 0.52). Kit foxes were more investigative than coyotes (difference in means = 0.07, 85% CI = 0.03–0.12), and both species were more investigative on protected lands than unprotected lands (difference in means = 0.08, 85% CI = 0.03–0.12).

## Discussion

We observed behavioral differences between coyotes and kit foxes suggesting coyotes were the more wary species, consistent with our predictions. Coyotes maintained a greater distance from novel stimuli and interacted with stimuli (e.g., biting, urinating, defecating on scent) less often than kit foxes. Coyotes increased averseness towards novel stimuli in unprotected areas, supporting our predictions. While anthropogenic activity still occurs on protected land, levels of recreation did not substantially influence surrounding wildlife [53]. Our results are consistent with previous research describing heightened aversiveness of coyotes to anthropogenic pressure [9].

Kit foxes were more also investigative in protected areas than unprotected, suggesting that increased anthropogenic pressure may result in increased neophobia. Overall, kit foxes were more investigate and maintained a closer distance to stimuli than coyotes. Contrasting histories of anthropogenic pressure may cause differences in behavior between species. However, the observed differences between species may be caused by other factors, as well. Underlying differences in social structure and landscape use may also affect behavior [25, 54]. Additionally, coyotes often represent the leading source of mortality for kit foxes [13, 55] and as such, coyote activity can influence habitat use and detection probability of kit foxes [12]. Additionally, individual personality and past experiences with anthropogenic stimuli may impact behavioral responses [4].

Numerous factors may influence the exploratory behavior of canids. Differences in behavior between individuals may have been related to social status or trophic level. Socially dominant coyotes were less neophobic in captivity; however, these characteristics may be selected against in the wild through predator control [1]. Higher trophic levels were associated with decreased neophobia [2]; however, we found that coyotes interacted with novel objects less than kit fox. Familiarity of the areas and levels of disturbance may also have influenced exploratory behavior. Coyotes in unfamiliar areas showed decreased neophobia compared to areas within their home range [6]. Spotted hyenas (*Crocuta crocuta*) exhibited less neophobia in areas with high levels of anthropogenic disturbance [5]. Developmental differences between species may also influence interactions with novel stimuli. Differences in motor skills and developmental trajectories led to wolves (*C*. *lupus*) interacting with novel objects and environments more than dogs [C. l. familiaris; 56]; however, habituation has led to decreased neophobia in dogs but not wolves [57]. Size of the object and duration of exposure can also influence the extent of exploratory behavior by canids. Coyotes interacted with smaller novel objects more than large objects, however, this effect reversed after objects were removed [24]. Similarly, Culpeo fox (*Lycocalopex culpaeus*) and grey foxes (*Urocyon cinereoargenteus*) increased exploration after novel objects were removed, despite initial neophobic responses from culpeo foxes [58]. Prior studies have highlighted the complexity of factors governing behavioral responses of canids to novel objects.

Anthropogenic pressures can affect various behaviors including mating, survival, social structure, and foraging of wildlife [7], often leading to increased wariness of anthropogenic stimuli [9, 59]. We provide additional research on the behavior of coyotes and kit foxes, highlighting behavioral differences between species in areas with and without hunting/trapping. Both species were more investigative on protected land than unprotected land. Coyotes maintained a greater distance from novel objects and were generally less investigative than kit foxes, potentially due to extensive exploitation causing a general increase in wariness of anthropogenic objects [4]. Our findings provide a behavioral basis for the commonly held notion that coyotes are more difficult to trap. Kit foxes were more investigative than coyotes, particularly on protected land, suggesting a greater sensitivity to anthropogenic pressure than coyotes. As kit foxes are a species of conservation concern, these results may be relevant to management efforts in areas of high disturbance. Our findings provide additional evidence that anthropogenic pressure can alter the fine-scale behavior of wildlife species.

## References

[1] Mettler AE, Shivik JA. Dominance and neophobia in coyote (Canis latrans) breeding pairs. Appl Anim Behav Sci. 2007;102(1):85–94.

[2] Crane AL, Ferrari MCO. Patterns of predator neophobia: a meta-analytic review. Proc Biol Sci. 2017;284(1861):20170583 10.1098/rspb.2017.0583 28835552 PMC5577474

[3] Bremner-Harrison S, Cypher BL, Van Horn Job C, Harrison SWR. Assessing personality in San Joaquin kit fox in situ: efficacy of field-based experimental methods and implications for conservation management. J Ethol. 2018;36(1):23–33. 10.1007/s10164-017-0525-9 29353954 PMC5746588

[4] Barrett B, Zepeda E, Pollack L, Munson A, Sih A. Counter-culture: Does social learning help or hinder adaptive response to human-induced rapid environmental change? Frontiers in Ecology and Evolution. 2019;7(183).

[5] Greenberg JR. Human disturbance affects personality development in a wild carnivore. Anim Behav. 2017;v. 132:pp. 303–12-2017 v.132.

[6] Harris CE, Knowlton FF. Differential responses of coyotes to novel stimuli in familiar and unfamiliar settings. Can J Zool. 2001;79(11):2005–13.

[7] Verdade LM. The influence of hunting pressure on the social behavior of vertebrates. Rev Bras Biol. 1996;56(1):1–13. 8731558

[8] Knight RL, Cole DN. Wildlife and recreationists: coexistence through management and research. Island Press, Washington, DC 1995 393 p.

[9] Kitchen AM, Gese EM, Schauster ER. Changes in coyote activity patterns due to reduced exposure to human persecution. Can J Zool. 2000;78:853–7.

[10] Reznick DN, Butler MJ, Rodd FH, Ross P. Life-history evolution in guppies (*Poecilia reticulata*) 6. Differential mortality as a mechanism for natural selection. Evolution. 1996;50(4):1651–60. 10.1111/j.1558-5646.1996.tb03937.x 28565709

[11] Moehrenschlager A, List R, Macdonald DW. Escaping intraguild predation: Mexican kit foxes survive while coyotes and golden eagles kill Canadian swift foxes. J Mammal. 2007;88(4):1029–39.

[12] Lonsinger RC, Gese EM, Bailey LL, Waits LP. The roles of habitat and intraguild predation by coyotes on the spatial dynamics of kit foxes. Ecosphere. 2017;8(3):e01749.

[13] Kozlowski AJ, Gese EM, Arjo WM. Niche overlap and resource partitioning between sympatric kit foxes and coyotes in the Great Basin Desert of western Utah. Am Midl Nat. 2008;160(1):191–208.

[14] Bekoff M. Coyote, *Canis latrans* In: Chapman JA, Feldhamer GA, editors. Wild Mammals of North America: Biology, Management, and Economics. Baltimore, MD: Johns Hopkins University Press; 1982 p. 447–59.

[15] Connolly GE. Predator control and coyote populations: a review of simulation models. In: Bekoff M, editor. Coyotes: Biology, Behaviour, and Management 1978 p. 327–45.

[16] Evans GD, Pearson EW. Federal coyote control methods used in the western United States, 1971–77. Wildl Soc Bull. 1980;8(1):34–9.

[17] Gier HT. Ecology and behaviour of the coyote (Canis latrans). In: Fox MW, editor. The Wild Canids 1975 p. 247–62.

[18] Voigt DR, Berg WE. Coyote. In: Novak M, Baker JA, Obbard ME, Malloch B, editors. Wild Furbearer Management and Conservation in North America. Ontario, Canada: Queen’s Printer for Ontario; 1999 p. 344–57.

[19] Sterner RT, Shumake A. Coyote damage-control research: a review and analysis. In: Bekoff M, editor. Coyotes: Biology, Behaviour, and Management 1978 p. 297–325.

[20] Andelt WF, Mahan BR. Behavior of an urban coyote. Am Midl Nat. 1980;103(2):399–400.

[21] Berentsen AR, Schmidt RH, Timm RM. Repeated exposure of coyotes to the coyote lure operative device. Wildl Soc Bull. 2006;34(3):809–14.

[22] Egoscue HJ. Ecology and life history of the kit fox in Tooele County, Utah. Ecology. 1962;43(3):481–97.

[23] Gompper ME, Roland WK, Ray JC, Lapoint SD, Bogan DA, Jason RC. A comparison of noninvasive techniques to survey carnivore communities in northeastern North America. Wildl Soc Bull. 2006;34(4):1142–51.

[24] Heffernan DJ, Andelt WF, Shivik JA. Coyote investigative behavior following removal of novel stimuli. J Wildl Manag. 2007;71(2):587–93.

[25] Larrucea ESQ, Brussard PF, Jaegar MM, Barrett RH. Cameras, coyotes, and the assumption of equal detectability. J Wildl Manag. 2007;71(5):1682–9.

[26] McClennen N, Wigglesworth RR, Anderson SH, Wachob DG. The effect of suburban and agricultural development on the activity patterns of coyotes (*Canis Latrans*). Am Midl Nat. 2001;146(1):27–36.

[27] Séquin ES. The influence of social status on coyote vulnerability to photo-capture [Thesis]. Nevada: University of Nevada, Reno; 2001.

[28] Séquin ES, Jaeger MM, Brussard PF, Barrett RH. Wariness of coyotes to camera traps relative to social status and territory boundaries. Can J Zool. 2003;81(12):2015–25.

[29] Windberg LA. Coyote responses to visual and olfactory stimuli related to familiarity with an area. Can J Zool. 1996;74(12):2248–53.

[30] Arjo WM, Gese EM, Bennett TJ, Kozlowski AJ. Changes in kit fox-coyote-prey relationships in the Great Basin Desert, Utah. West N Am Nat. 2007;67(3):389–401.

[31] Cypher BL, Warrick GD, Otten MRM, O'Farrell TP, Berry WH, Harris CE, et al Population dynamics of San Joaquin kit foxes at the Naval Petroleum Reserves in California. Wildl Monogr. 2000;145:1–43.

[32] Haight RG, Cypher B, Kelly PA, Phillips S, Ralls K, Possingham HP. Optimizing reserve expansion for disjunct populations of San Joaquin kit fox. Biol Conserv. 2004;117(1):61–72.

[33] U.S. Fish and Wildlife Service. Recovery plan for upland species of the San Joaquin Valley, California. Region 1, Portland, OR: 1998.

[34] Zoellick BW, Smith NS. Size and spatial organization of home ranges of kit foxes in Arizona. J Mammal. 1992;73(1):83–8.

[35] Egoscue HJ. Preliminary studies of the kit fox in Utah. J Mammal. 1956;37(3):351–7.

[36] Wauer RH. Peculiar actions of coyote and kit fox. J Mammal. 1961;42:109–.

[37] Clark HO Jr. Marking of novel objects by kit foxes. Calif Fish Game. 2007;93(2):103–6.

[38] Kluever BM, Gese EM, Dempsey SJ, Knight RN. A comparison of methods for monitoring kit foxes at den sites. Wildl Soc Bull. 2013;37(2):439–43.

[39] McGrew JC. Distribution and habitat characteristics of the kit fox (*Vulpes macrotis*) in Utah [Thesis]. Logan: Utah State University; 1977.

[40] Thacker RK, Flinders JT. Kit or swift fox, *Vulpes velox*. In: Wilson DE, Ruff S, editors. The Smithsonian Book of North American Mammals Washington, D.C: Smithsonian Institution Press; 1999 p. 148–50.

[41] Fitzgerald JP, Meaney CA, Armstrong DM. Mammals of Colorado: University Press of Colorado; 1994 467 p.

[42] Hall LK, Larsen RT, Knight RN, Bunnell KD, McMillan BR. Water developments and canids in two North American deserts: a test of the indirect effect of water hypothesis. PLOS ONE. 2013;8(7):e67800 10.1371/journal.pone.0067800 23844097 PMC3699512

[43] Bowman J. Is dispersal distance of birds proportional to territory size? Can J Zool. 2003;81(2):195.

[44] Bowman J, Jaeger JAG, Fahrig L. Dispersal distance of mammals is proportional to home range size. Ecology. 2002;83(7):2049–55.

[45] Nelson JL, Cypher BL, Bjurlin CD, Creel S. Effects of habitat on competition between kit foxes and coyotes. J Wildl Manag. 2007;71(5):1467–75.

[46] Richards KA. Optimizing efforts to monitor kit foxes (*Vulpes macrotis*) in Utah. Provo, UT: Brigham Young University; 2017.

[47] LANDFIRE Existing Vegetation Type layer [Internet]. 2013.

[48] Bates D, Maechler M, Bolker BM, Walker SC. Fitting linear mixed-effects models using lme4. Journal of Statistical Software. 2015;67(1):1–48.

[49] R Development Core Team. R: a language and environment for statistical computing. Vienna, Austria: R Foundation for Statistical Computing; 2015.

[50] Liang H, Wu H, Zou G. A note on conditional AIC for linear mixed-effect models. Biometrika. 2008;95(3):773–8. 10.1093/biomet/asn023 19122890 PMC2572765

[51] Saefken B, Ruegamer D, Kneib T, Greven S. “Conditional model selection in mixed-effects models with cAIC4.” 2018; *ArXiv e-prints*. 1803.05664.

[52] Arnold TW. Uninformative parameters and model selection using Akaike's Information Criterion. J Wildl Manag. 2010;74(6):1175–8.

[53] Kays R, Parsons AW, Baker MC, Kalies EL, Forrester T, Costello R. Does hunting or hiking affect wildlife communities in protected areas? J Appl Ecol. 2017;54(1):242–52.

[54] Kitchen AM, Gese EM, Schauster ER. Resource partitioning between coyotes and swift foxes: space, time, and diet. Can J Zool. 1999;77(10):1645–56.

[55] Cypher BL, Spencer KA. Competitive interactions between coyotes and San Joaquin kit foxes. J Mammal. 1998;79(1):204–14.

[56] Marshall-Pescini S, Schwarz JFL, Kostelnik I, Virányi Z, Range F. Importance of a species’ socioecology: Wolves outperform dogs in a conspecific cooperation task. Proc Natl Acad Sci USA. 2017;114(44):11793–8. 10.1073/pnas.1709027114 29078337 PMC5676910

[57] Wheat CH, van der Bijl W, Temrin H. Dogs, but not wolves, lose their sensitivity toward novelty with age. Frontiers in Psychology. 2019;10(2001):1–12. 31555182 10.3389/fpsyg.2019.02001 PMC6742907

[58] Travaini A, Vassallo AI, García GO, Echeverría AI, Zapata SC, Nielsen S. Evaluation of neophobia and its potential impact upon predator control techniques: A study on two sympatric foxes in southern Patagonia. Behav Process. 2013;92:79–87. 10.1016/j.beproc.2012.10.008 23124014

[59] Cypher BL, Scrivner JH. Coyote control to protect endangered San Joaquin kit foxes at the Naval Petroleum Reserves, California. Proceedings of the Vertebrate Pest Conference. 1992;15:42–7.

