# Supplementary material for: Behavioral differences at scent stations between two exploited species of desert canids
Source: PLoS One. 2020 May 15;15(5):e0232492. doi: 10.1371/journal.pone.0232492 (PMC7228668; doi:10.1371/journal.pone.0232492)
Supplement: Attachment — Submitted filename: Response_to_Reviewers.docx [file pone.0232492.s001.docx]

Journal Requirements:

When submitting your revision, we need you to address these additional requirements.

1) Please ensure that your manuscript meets PLOS ONE's style requirements, including those for file naming. The PLOS ONE style templates can be found at

<http://www.journals.plos.org/plosone/s/file?id=wjVg/PLOSOne_formatting_sample_main_body.pdf> and <http://www.journals.plos.org/plosone/s/file?id=ba62/PLOSOne_formatting_sample_title_authors_affiliations.pdf>

*We have edited the formatting to ensure compliance with the PLOS One guidelines.*

2) In your Methods section, please provide additional location information of the study areas, including geographic coordinates for the data set if available.

*While we cannot share the exact locations of the camera sites, we have added some additional text to the methods section describing the study area and have included a reference to the map of the study area, pinpointing the study sites within the state of Utah.*

3) Please include your tables as part of your main manuscript and remove the individual files. Please note that supplementary tables (should remain/ be uploaded) as separate "supporting information" files

*We have included our model selection table at the end of the manuscript as opposed to uploading it as a separate file.*

4)  We note that you have indicated that data from this study are available upon request. PLOS only allows data to be available upon request if there are legal or ethical restrictions on sharing data publicly. For more information on unacceptable data access restrictions, please see <http://journals.plos.org/plosone/s/data-availability#loc-unacceptable-data-access-restrictions>.

In your revised cover letter, please address the following prompts:

a) If there are ethical or legal restrictions on sharing a de-identified data set, please explain them in detail (e.g., data contain potentially sensitive information, data are owned by a third-party organization, etc.) and who has imposed them (e.g., an ethics committee). Please also provide contact information for a data access committee, ethics committee, or other institutional body to which data requests may be sent.

b) If there are no restrictions, please upload the minimal anonymized data set necessary to replicate your study findings as either Supporting Information files or to a stable, public repository and provide us with the relevant URLs, DOIs, or accession numbers. For a list of acceptable repositories, please see <http://journals.plos.org/plosone/s/data-availability#loc-recommended-repositories>.

We will update your Data Availability statement on your behalf to reflect the information you provide.

*We have made the data available online in a Github repository, the link is as follows:*

<https://github.com/MaksimSergeyev/CanidBehaviorScentStations>

5) Thank you for stating the following in the Acknowledgments Section of your manuscript:

[We are grateful to Bureau of Land Management, Utah Department of Natural Resources,
US Army Dugway Proving Ground, US Hill Air Force Base, and Brigham Young University for
funding this research and thank our technicians for their assistance.]

We note that you have provided funding information that is not currently declared in your Funding Statement. However, funding information should not appear in the Acknowledgments section or other areas of your manuscript. We will only publish funding information present in the Funding Statement section of the online submission form.

Please remove any funding-related text from the manuscript and let us know how you would like to update your Funding Statement. Currently, your Funding Statement reads as follows:

 [The author(s) received no specific funding for this work.]

Please include the updated Funding Statement in your cover letter. We will change the online submission form on your behalf.

*We have edited the Acknowledgements section to remove any information pertaining to funding and have edited the funding statement in the submission process to include the funding previously mentioned in the manuscript.*

Reviewer #1: This is a very interesting study in which the authors tried to explore the different behavioral responses of coyotes and kit foxes to novel objects within and outside the protected area by infra-cameras. The idea of this study is innovative and sharp. The manuscript is short, simple, and well written. The results are promising. I have no further comments and questions about its acceptance except the following questions:

1) It is good to compare the two species’ behavioral responses to novel objects within and outside the protected area, and you have provided a criteria of 2.6km radius buffer based on the home ranges of the two species. However, I am thinking the dispersal distance of each species might be more important than that of the home range/daily movement, due to the fact that you are studying the long-term effects/pressures of anthropogenic activities. Thus, the grouping of putting the site “Thomas Range” into unprotected area (if my understanding is right) might be a problem. I would suggest you re-analysis your data by excluding this part of data from your whole data set to see if you still get the same result. Then, you can soundly make the statement.

*We agree with this comment and have removed the ‘Thomas Range’ data set from the analysis and reran the models. We updated the Results and figures (Fig. 1 and 3) to reflect these changes and changed some text in the Discussion to describe the new results.*

2) Additional information about the two technicians may be needed for categorizing photographs as close/far and investigative or non-investigative. Did they clearly know the experimental design and grouping of the scents in each photograph? How did you manage to reduce the observer’s bias in doing so?

*We added some additional language to the methods section to clarify that observers were familiar with the study design and trained to classify photographs. We reduced bias by having all photographs classified as close/far by one individual and then investigative/non-investigative by a second individual and then assessed for quality afterwards.*

3) You mentioned in the results section (Line 5 on page 8)” We identified trends suggesting coyotes were warier on protected lands than unprotected land, however these trends were not statistically significant and were consistent for both species”. What does this mean or what are the possible causes for this result? It might be better for readers to understand to provide some explanations in the discussion section.

*We edited much of the text in the Results section after rerunning the analysis without the ‘Thomas Range’ data set. After revision, this sentence no longer appears in the text.*
